# CD4^+^CD8^+^ T follicular helper cells regulate humoral immunity in chronic inflammatory lesions

**DOI:** 10.3389/fimmu.2022.941385

**Published:** 2022-08-25

**Authors:** Kosuke Murayama, Ippei Ikegami, Ryuta Kamekura, Hiroshi Sakamoto, Masahiro Yanagi, Shiori Kamiya, Taiki Sato, Akinori Sato, Katsunori Shigehara, Motohisa Yamamoto, Hiroki Takahashi, Ken-ichi Takano, Shingo Ichimiya

**Affiliations:** ^1^ Department of Human Immunology, Research Institute for Frontier Medicine, Sapporo Medical University School of Medicine, Sapporo, Japan; ^2^ Department of Otolaryngology and Head and Neck Surgery, Sapporo Medical University School of Medicine, Sapporo, Japan; ^3^ Department of Rheumatology and Allergy, IMSUT Hospital, Institute of Medical Science, The University of Tokyo, Tokyo, Japan; ^4^ Department of Clinical Immunology and Rheumatology, Sapporo Medical University School of Medicine, Sapporo, Japan

**Keywords:** DP-Tfh cells, memory B cells, chronic inflammation, IgG4-RD, tonsils

## Abstract

T follicular helper (Tfh) cells drive humoral immunity by facilitating B cell responses at the initial and recall phases. Recent studies have indicated the possible involvement of Tfh cells in the process of chronic inflammation. However, the functional role of Tfh cells in persistent immune settings remains unclear. Here, we report that CD4^+^CD8^+^ (double-positive, DP; CD3^+^CD4^+^CD8^+^CXCR5^hi^PD-1^hi^) Tfh cells, a subset of germinal-center-type Tfh cells, were abundantly present in the fibroinflammatory lesions of patients with immunoglobulin G4-related disease (IgG4-RD). Transcriptome analyses showed that these DP-Tfh cells in the lesions of IgG4-RD preferentially expressed signature genes characteristic of cytotoxic CD8^+^ T cells, such as Eomes, CRTAM, GPR56, and granzymes, in addition to CD70. Scatter diagram analyses to examine the relationships between tissue-resident lymphocytes and various clinical parameters revealed that the levels of DP-Tfh cells were inversely correlated to the levels of serum IgG4 and local IgG4-expressing (IgG4^+^) memory B cells (CD19^+^CD27^+^IgD^-^) in patients with IgG4-RD. Cell culture experiments using autologous tonsillar lymphocytes further suggested that DP-Tfh cells possess a poor B-cell helper function and instead regulate memory B cells. Since CD4^+^ (single positive, SP; CD3^+^CD4^+^CD8^-^CXCR5^hi^PD-1^hi^) Tfh cells differentiated into DP-Tfh cells under stimulation with IL-2 and IL-7 as assessed by *in vitro* experiments, these data imply that SP-Tfh cells are a possible origin of DP-Tfh cells under persistent inflammation. These findings highlight the potential feedback loop mechanism of Tfh cells in immune tolerance under chronic inflammatory conditions. Further studies on DP-Tfh cells may facilitate control of unresolved humoral responses in IgG4-RD pathological inflammation.

## Introduction

CD4^+^ T helper cells as well as their diverse distinct subsets drive humoral and cellular immune responses for host defense under pathological conditions (1). Humoral immunity plays a cardinal role in immune responses, therefore, the T follicular helper cells (Tfh cells) which are an effector subset of helper CD4^+^ T cells, have received much interest (2, 3). Tfh cells play an essential role in facilitating germinal center (GC) formation by B cells to generate high-affinity antibodies as well as long-lived plasma cells and memory B cells. The antagonistic functions of the two transcription factors, B-cell lymphoma 6 (Bcl6), which is a master regulator of Tfh cells, and B lymphocyte-induced maturation protein 1 (Blimp1), toward each other during the development of Tfh cells from naïve CD4^+^ T cells underlie a mechanism for bimodal cell fate decision-making to specify Tfh cell identity among effector helper CD4^+^ T cells (4). The productive interactions of Tfh and B cells are controlled by the C-X-C motif chemokine receptor 5 (CXCR5) and sphingosine-1 phosphate receptor 2 (S1PR2) expressed on Tfh cells, which are responsible for the effective distribution of Tfh cells to tissues to enable their cognate interactions with B cells (5). After activation of Tfh cells in lymphoid tissues, a portion of tissue-resident Tfh cells enters the systemic circulation as memory-like Tfh cells (Tfh1, Tfh2, and Tfh17 cells) with the functional feature of a class switch recombination for B-cell activation (6). In these contexts, studies on Tfh cells and their related lymphocytes can renew the landscape of Tfh cell-mediated humoral immunity in immunocompetent settings as well as in the aberrant immune responses underlying pathological chronic inflammation (7, 8).

Immunoglobulin G4-related disease (IgG4-RD) belongs to a unique category of chronic immune-mediated diseases, that are primarily recognized as an archetypal Mikulicz’s disease of IgG4-related dacryoadenitis and sialadenitis (9, 10). IgG4-RD occurs in multiple organs including salivary glands, lacrimal glands, pancreas, thyroid glands, lung, biliary tree, and retroperitoneum, and is characterized by the presence of high serum levels of IgG4. In tissue lesions of IgG4-RD, ectopic GCs are observed as a common histopathological feature in addition to marked infiltration of IgG4-positive B cells, storiform-pattern fibrosis, and obliterative phlebitis. While steroid therapy is effective in the treatment of IgG4-RD, relapse occasionally occurs after dose tapering or intermittent administration of glucocorticoids (11). Although the pathogenesis of IgG4-RD remains controversial, rituximab, an anti-CD20 antibody that decreases B-cell activity, is generally beneficial for IgG4-RD treatment, suggesting that B cells are the target cells of steroid therapy for IgG4-RD (12). In this regard, dysregulation of B-cell-mediated immunity is thought to be related to the pathogenesis of IgG4-RD. Accumulating evidence has shown that the tissue lesions of IgG4-RD preferentially harbor Tfh cells (13, 14); however, an understanding of the functional role of Tfh cells in B-cell regulation and its mechanism of action within the inflammatory environment of IgG4-RD has been a topic of debate in recent years.

To address the possible role of Tfh cells in IgG4-RD, we investigated Tfh cells and other lymphocyte subsets in tissue lesions of IgG4-RD in this study. Ectopic GCs are commonly formed in IgG4-RD lesions; therefore, we focused on GC-type Tfh cells (CD3^+^CD4^+^CXCR5^hi^PD-1^hi^) and comprehensively analyzed their transcriptome (15). In comparison with GC-type Tfh cells in tonsils, GC-type Tfh cells in the inflammatory lesions of IgG4-RD preferentially expressed CD8 (CD3^+^CD4^+^CD8^+^CXCR5^hi^PD-1^hi^). These cells are thus referred to as CD4^+^CD8^+^ (double-positive; DP) Tfh cells. The CD8 expression in DP-Tfh cells was lower than that in canonical CD8^+^ cytotoxic T cells (CTLs). However, DP-Tfh cells expressed CTL-related signature genes such as eomesodermin (Eomes), class I-restricted T cell-associated molecule (CRTAM), G protein-coupled receptor 56 (GPR56), perforin, and granzymes (16, 17). Interestingly, clinical studies of DP-Tfh cells in IgG4-RD and *in vitro* experiments using autologous lymphocytes of tonsils have suggested that memory B cells are a potential target of DP-Tfh cells. Since GC-type Tfh cells in tonsils upregulate CD8 upon stimulation with IL-2 and IL-7, which are usually associated with the maintenance of T cell activation and memory T cells, authentic CD4^+^ GC-type Tfh cells may be able to generate DP-Tfh cells to regulate surrounding memory B cells under inflammatory conditions. By focusing on the functional and developmental processes of DP-Tfh cells, further studies could provide a novel modality to resolve undesirable immune responses in IgG4-RD and other pathological conditions underlying chronic inflammation.

## Materials and methods

### Study populations

The study populations are summarized in **Supplementary Table S1**. Diagnosis of IgG4-RD was performed in accordance with widely recognized diagnostic criteria (18). The type of organ involved in IgG4-RD patients is summarized in **Supplementary Table S2**. None of the recruited patients had received standard glucocorticoid therapy before surgical resection of submandibular glands (SMGs).

### Tissues

Tissues from SMGs and palatine tonsils were obtained from patients with IgG4-RD and tonsillar hypertrophy for diagnosis or treatment at Sapporo Medical University Hospital, Japan. Cells in tissues were analyzed by flow cytometry and *in vitro* studies, and a portion of the tissues was employed to prepare formalin-fixed paraffin-embedded (FFPE) sections.

### Antibodies and other reagents

Antibodies for flow cytometry and immunohistochemistry as well as reagents used for *in vitro* studies are summarized in **Supplementary Table S3**.

### Flow cytometry and cell sorting

Tissues were mechanically disrupted and lymphocytes in single-cell suspensions were prepared by density-gradient centrifugation with Lympholyte-H (Cedarlane, Burlington, Canada). Then, the cells were stained with antibodies to determine the expression of specific molecules using flow cytometry. The cells were analyzed or sorted using a FACS Canto II or FACS Aria II and III (BD Biosciences, New Jersey, USA) in combination with magnetic bead sorting (Miltenyi Biotec, North Rhine-Westphalia, Germany). In each experiment, samples were analyzed for singlet events with doublet discrimination. The purity of FACS-sorted cells reached 95% after validation by reanalysis using the FACS Canto II. The flow cytometry data were analyzed using FACS DiVA and FlowJo software (BD Biosciences).

### Microarray analysis

Total RNA was extracted using TRIzol reagent (Life Technologies, California, USA) and validated with a 2100 Bioanalyzer (Agilent Technologies, California, USA) and NanoDrop microvolume spectrophotometer (Thermo Fisher Scientific, Massachusetts, USA). Then, the RNA was amplified and labelled with Cy3-CTP to obtain cRNA using a Quick Amp Labelling kit (Agilent Technologies) and then hybridized to a microarray plate (SurePrint G3 Human GE 8×60K v3; Agilent Technologies). The obtained data were analyzed by bioinformatics software (Riken Genesis, Tokyo, Japan) and Heatmapper software (The University of Alberta, Alberta, Canada). Data were further investigated by gene set enrichment analysis (GSEA v2.0.13 software, UC San Diego, California, USA) and the iPathwayGuide platform (Advaita Bioinformatics, Ann Arbor, Michigan, USA).

### RT-qPCR analysis

First-strand cDNA was synthesized from total RNA by using a High-Capacity cDNA Reverse Transcription kit (Thermo Fisher Scientific). Quantitative PCR analysis was conducted to detect gene-specific products using SYBR green and TaqMan probes with the Light Cycler 96 System (Roche, Basel, Switzerland). The PCR primer pairs and probes used are summarized in **Supplementary Table S4**.

### Immunohistochemistry

FFPE tissue sections were immunestained using a standard protocol to detect IL-7. After staining, signals were visualized with 3,3′-diaminobenzidine and the sections were counterstained with hematoxylin. For histological differentiation, corresponding tissue sections were stained with hematoxylin and eosin.

### Transmission electron microscopy

Transmission electron microscopy was performed using a standard protocol. Ultrathin sections were prepared using an ultramicrotome, mounted on a copper grid, and examined under a transmission electron microscope (JEM-1400; JEOL, Tokyo, Japan).

### Cell culture experiments

FACS-sorted tonsillar lymphocytes were used for cell culture experiments in a humidified atmosphere with 5% CO_2_ at 37°C. For co-culture of T and B cells, DP-Tfh or SP-Tfh cells were seeded in a 96-well round-bottom plate with autologous B cells at a 1:1 ratio (5×10^4^ cells/well) in 200 μL of AIM-V medium containing 2 μg/mL anti-CD3 mAb, 2 μg/mL anti-CD28 mAb, and 1 μg/mL CD40L. After incubation for 7 days, the supernatants were analyzed using an ELISA kit to measure IgG (R&D Systems, Minneapolis, USA) and a cytotoxicity LDH assay kit-WST (Dojindo, Tokyo, Japan) to evaluate cytotoxicity. To examine the secretion of cytotoxic granules, cells (5 × 10^4^ cells) were seeded in a 96-well plate in 200 μL of AIM-V medium containing 2 μg/mL anti-CD3 mAb and 2 μg/mL anti-CD28 mAb, and incubated for 7 days. Subsequently, the supernatants were analyzed by an ELISA kit for granzyme B (R&D Systems).

### Statistical analyses

Results are expressed as mean ± SEM. Statistical analyses were performed using Mann–Whitney U test or Spearman’s rank correlation test, where applicable. In the all analyses, *p* < 0.05 was considered significant (*p < 0.05, **p < 0.01, ***p < 0.001, ****p < 0.0001), while *p* > 0.05 was considered non-significant (n.s.). The statistical tests were performed using GraphPad Prism 8 software (GraphPad, San Diego, CA).

## Results

### Tfh cells in IgG4-RD lesions have a cytotoxic phenotype

**Figure 1:**
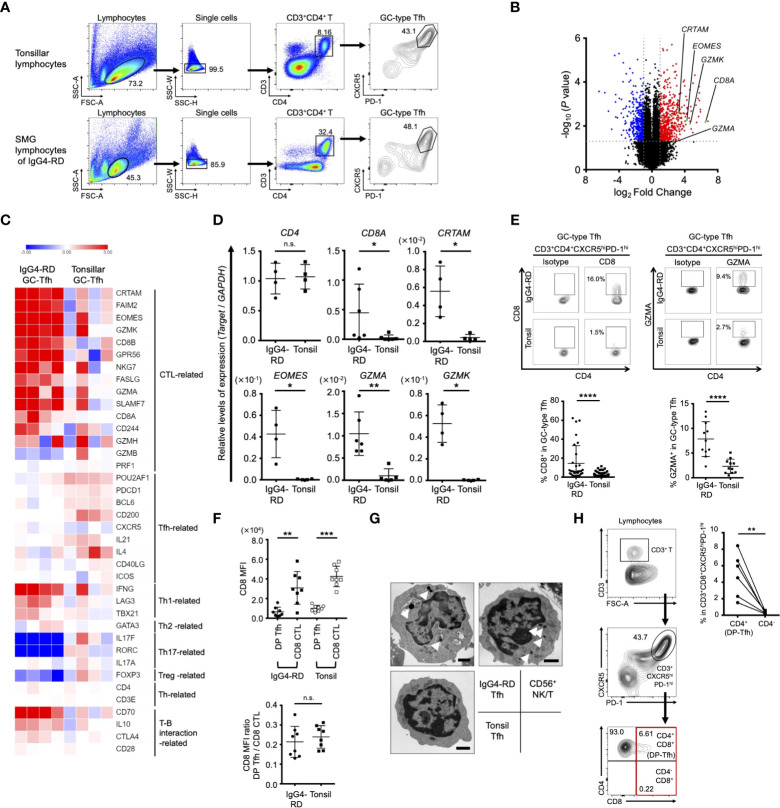
Phenotypic characteristics of GC-type Tfh cells in IgG4-RD lesions. **(A)** Flow cytometry profiles showing selected windows and gating strategy applied to identify GC-type Tfh cells (CD3^+^CD4^+^CXCR5^hi^PD-1^hi^) in lymphocytes of tonsils and SMG lesions of IgG4-RD. **(B)** Volcano plot identifying differentially expressed genes (*p* < 0.05) with more than two-fold expression in GC-type Tfh cells localized in SMG lesions of IgG4-RD versus GC-type Tfh cells in tonsils. The red and blue dots indicate upregulated and downregulated genes, respectively, in the Tfh cells of IgG4-RD. Data were obtained from microarray analysis of four specimens in each experiment group of IgG4-RD lesions or tonsils. **(C)** Heat map indicating relative abundances of transcripts identified in **(A)** for selected genes regulating helper CD4^+^ T cells and CTLs. Relative values of gene expression are indicated by color. **(D)** Relative expression levels of genes in GC-type Tfh cells in the SMG lesions of IgG4-RD and tonsils as indicated in **(C)** assessed by RT-qPCR analysis. GAPDH was used as a control (IgG4-RD, n = 4-6; tonsil, n = 4-6). **(E)** Representative flow cytometry profiles of the expression of CD8 and granzyme A (GZMA) in GC-type Tfh cells in SMG lesions of IgG4-RD and tonsils (upper panels). Graphs indicating the expression of CD8 (IgG4-RD, n = 31; tonsil, n = 71) and GZMA (IgG4-RD, n = 11; tonsil, n = 11) in GC-type Tfh cells as assessed by flow cytometry (lower panels). **(F)** Expression levels of CD8 on DP-Tfh cells and CD8^+^ CTLs in the lymphocytes of IgG4-RD lesions and tonsils (IgG4-RD, n = 8; tonsil, n = 8) assessed by flow cytometry. MFI, mean fluorescence intensity. **(G)** Transmission electron microscopy of FACS-sorted T cells, including GC-type Tfh cells from SMG lesions of IgG4-RD and tonsils and NKT cells (CD3^+^CD56^+^) from tonsils. Arrowheads indicate electron-dense granules in the cytosol. Scale bar: 1 μm. **(H)** DP-Tfh cells enriched in the CD3^+^CD8^+^CXCR5^hi^PD-1^hi^ T cell population in tonsils. Representative flow cytometry profiles to detect DP-Tfh cells and CD3^+^CD4^-^CD8^+^CXCR5^hi^PD-1^hi^ T cells as indicated in the red square (left panels). A graph showing the CD3^+^CD8^+^CXCR5^hi^PD-1^hi^ T cell population preferentially containing DP-Tfh cells (right). Data from the same tonsils are paired (n = 6). Data in **(D–F, H)** were analyzed by the Mann–Whitney U test. Data in **(G)** were obtained from three independent experiments.

Following a previous study, which showed the abundance of tissue-resident Tfh cells (CD3^+^CD4^+^CXCR5^+^PD-1^+^) in SMG lesions in patients with IgG4-RD (14), we examined the gene expression profile of GC-type Tfh cells (CD3^+^CD4^+^CXCR5^hi^PD-1^hi^) in IgG4-RD lesions and compared it with that of GC-type Tfh cells in tonsils (**Figure 1A**). The results indicated that GC-type Tfh cells in IgG4-RD lesions significantly expressed signature genes of CTLs, including CD8, Eomes, CRTAM, and granzymes (**Figure 1B** and **Supplementary Table S5**). Further analysis of transcriptomes showed that other CTL-related genes, such as GPR56 and FAS ligand, were also upregulated in GC-type Tfh cells in the SMG lesions of IgG4-RD, whereas the expression levels of Tfh-related genes, such as IL-21, CD200, and Pou2af1 (also named as Bob1), in GC-type Tfh cells of IgG4-RD appeared to be relatively lower than those of GC-type Tfh cells in tonsils (**Figure 1C**). GC-type Tfh cells of IgG4-RD expressed low levels of genes related to Th2, Th17, and Treg cells, while genes related to Th1 cells and interactions of T cells with B cells such as CD70 were well expressed in these cells (19). Validation studies further showed that the GC-type Tfh cells in IgG4-RD lesions showed higher expression of CD8, Eomes, CRTAM, and granzymes in comparison with GC-type Tfh cells in tonsils (**Figures 1D, E**). Based on the expression profile of CD8, we here referred to CD8-expressing GC-type Tfh cells as CD4^+^CD8^+^ Tfh cells (double-positive Tfh cells, DP-Tfh cells; CD3^+^CD4^+^CD8^+^CXCR5^hi^PD-1^hi^). The frequency of DP-Tfh cells in IgG4-RD lesions ranged from 1.1% to 62.3% (average, 14.5% of the total GC-type Tfh cells; n = 31; **Figure 1E**). DP-Tfh cells were also detected in tonsils and constituted about 0.1%–11.5% of the total GC-type Tfh cells (average, 2.3%; n = 71; **Figure 1E**). CD8 protein expression on DP-Tfh cells was lower than that on canonical CD8^+^ CTLs in both IgG4-RD lesions and tonsils (**Figure 1F**). Additionally, CD8α and CD8β were almost equally expressed on DP-Tfh cells of IgG4-RD lesions and tonsils (data not shown). The ultrastructural analysis indicated the cytotoxic activity of GC-type Tfh cells, which possessed electron-dense granules similar to NK cells, but not of GC-type Tfh cells in tonsils (**Figure 1G**). Collectively, these data indicated that DP-Tfh cells with a possible CTL-like ability are frequently found in the GC-type Tfh cell population residing in IgG4-RD lesions. To a lesser extent, such DP-Tfh cells were detected among GC-type Tfh cells as a minor population in the tonsils. Most CD3^+^CD8^+^CXCR5^hi^PD-1^hi^ cells expressed CD4 in tonsillar lymphocytes as identified with DP-Tfh cells (**Figure 1H**).

### Clinical significance of DP-Tfh cells in IgG4-RD

**Figure 2:**
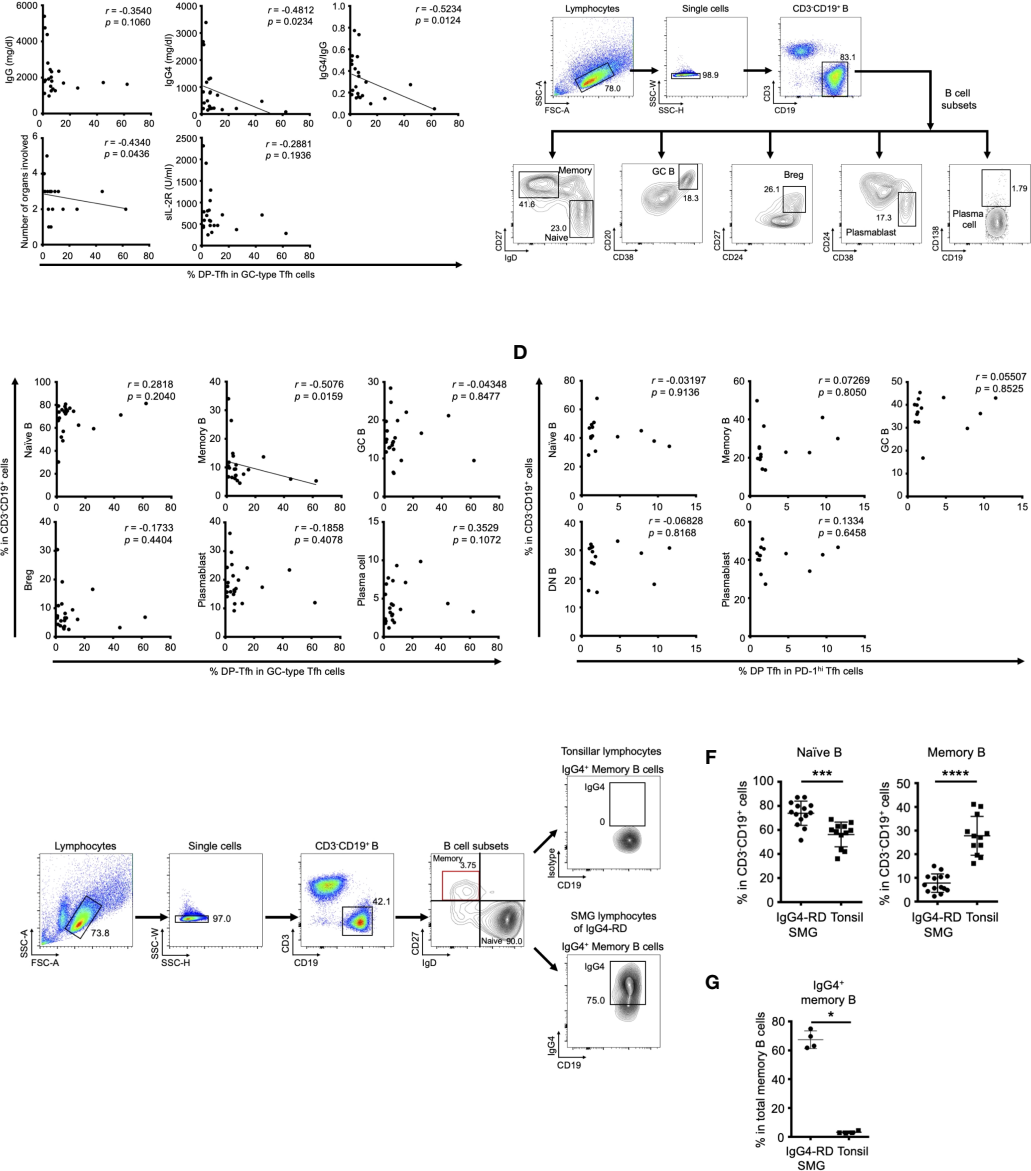
Clinical association of DP-Tfh cells in IgG4-RD lesions. **(A)** Scatter plots showing relationships between various clinical parameters associated with the IgG4-RD disease state and the ratios of DP-Tfh cells (CD3^+^CD4^+^CD8^+^CXCR5^hi^PD-1^hi^) to total GC-type Tfh cells (CD3^+^CD4^+^CXCR5^hi^PD-1^hi^) in SMG lesions of IgG4-RD (n = 22). **(B)** Flow cytometry profiles showing selected windows and gating strategies applied to identify B cells (CD3^-^CD19^+^) and B-cell subsets (naïve B cells, CD3^-^CD19^+^IgD^+^CD27^-^; memory B cells, CD3^-^CD19^+^IgD^-^CD27^+^; GC B cells, CD3^-^CD19^+^CD20^+^CD38^+^; regulatory B cells, CD3^-^CD19^+^CD24^hi^CD27^+^; plasmablasts, CD3^-^CD19^+^CD24^-^CD38^+^; plasma cells, CD3^-^CD19^+^CD138^+^) in SMG lesions of IgG4-RD. **(C)** Scatter plots indicating the relationships between the ratios of various B-cell subsets and ratios of DP-Tfh cells to total GC-type Tfh cells in SMG lesions of IgG4-RD (n = 22) measured by flow cytometry. **(D)** Scatter plots indicating relationships between the ratios of various B-cell subsets and ratios of DP-Tfh cells to total GC-type Tfh cells in tonsils (n = 14) measured by flow cytometry. **(E)** Flow cytometry profiles showing selected windows and gating strategy for IgG4-expressing memory B cells of lymphocytes of tonsils and SMG lesions of IgG4-RD. **(F)** Naïve and memory B cells in SMG lesions of IgG4-RD (n = 14) and tonsils (n = 12) analyzed by flow cytometry. **(G)** High frequency of memory B cells expressing IgG4 (IgG4^+^ memory B cells) in SMG lesions of IgG4-RD analyzed by flow cytometry (IgG4-RD, n = 4; tonsil, n = 4). Data in **(A, C, D)** were analyzed by the Spearman’s rank correlation test, and data in **(F, G)** were studied by the Mann–Whitney U test.

To address the functional role of DP-Tfh cells in the etiology of IgG4-RD, we analyzed scatter plot diagrams of the levels of DP-Tfh cells and various clinical parameters in patients with IgG4-RD. The results showed a marked inverse correlation of the levels of DP-Tfh cells with the serum IgG4 level (*r* = -0.4812, *p* = 0.0234), and the ratio of serum IgG4 to total IgG (*r* = -0.5234, *p* = 0.0124; **Figure 2A**). We also obtained similar results from scatter plot analysis of the level of DP-Tfh cells and the number of involved organs in IgG4-RD (*r* = -0.434, *p* = 0.0436; **Figure 2A** and **Supplementary Table S2**). Since the serum level of IgG4 is well associated with the disease severity of IgG4-RD (11), these findings imply a possible role of DP-Tfh cells in regulating a certain pathway(s) involved in the production of IgG4 in IgG4-RD. Next, we performed scatter chart analyses to determine the relationships between DP-Tfh cells and different B-cell subsets in IgG4-RD lesions (**Figure 2B**). Interestingly, the results indicated an inverse correlation between the levels of DP-Tfh cells and memory B cells (*r* = -0.5076, *p* = 0.0159; **Figure 2C**), which promptly and effectively induce a humoral recall response as antigen-experienced B cells (20). Conversely, the levels of other B-cell subsets, including naïve B cells, GC B cells, plasmablasts, plasma cells, and regulatory B cells, did not show any correlation to the level of DP-Tfh cells in the tissue lesions of IgG4-RD. Even though the SMG lesions of IgG4-RD with the formation of ectopic lymphoid structures could not fully harbor a memory B cell pool, IgG4-expressing (IgG4^+^) memory B cells were fairly enriched in the lesions (**Figures 2E–G**). Thus, DP-Tfh cells could be considered to have a potential role in regulating IgG4 production by controlling IgG4^+^ memory B cells in IgG4-RD lesions. While we did not find functional correlations between DP-Tfh cells and B-cell subsets residing in tonsils (**Figure 2D**), the clinical significance of DP-Tfh cells might be observed in ectopic lymphoid tissues under chronic inflammation rather than secondary lymphoid tissues.

### DP-Tfh cells vary from SP-Tfh cells

**Figure 3:**
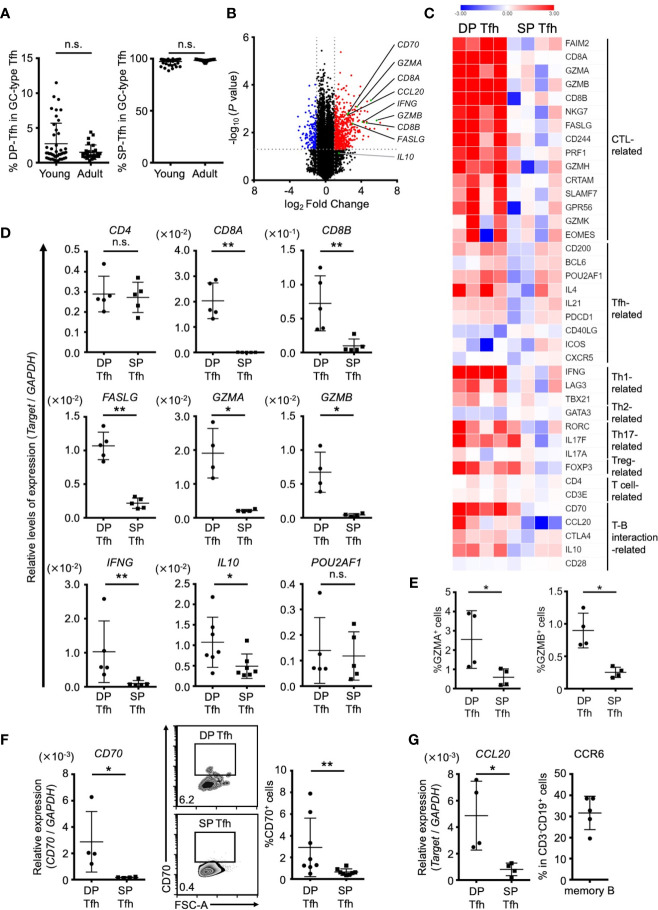
Different characteristics of DP-Tfh and SP-Tfh cells in tonsils. **(A)** Ratio of DP-Tfh cells (CD3^+^CD4^+^CD8^+^CXCR5^hi^PD-1^hi^) to total GC-type Tfh cells (CD3^+^CD4^+^CXCR5^hi^PD-1^hi^) and ratio of SP-Tfh cells (CD3^+^CD4^+^CD8^-^CXCR5^hi^PD-1^hi^) to total GC-type Tfh cells in young (n = 44) and adult (n = 27) tonsils assessed by flow cytometry are shown in the left and right, respectively. **(B)** Volcano plot showing differentially expressed genes (*p* < 0.05) with more than two-fold expression in DP-Tfh cells versus SP-Tfh cells (CD3^+^CD4^+^CD8^-^CXCR5^hi^PD-1^hi^) derived from tonsils. The red and blue dots indicate the upregulated and downregulated genes, respectively, in DP-Tfh cells. Data were obtained from microarray analysis of four tonsil specimens in each experimental group studying DP-Tfh and SP-Tfh cells. **(C)** Heat map representing the relative abundances of transcripts identified in **(B)** for selected genes regulating helper CD4^+^ T cells and CTLs. Relative values of gene expression are indicated by color. **(D)** Relative levels of expression of genes in DP-Tfh and SP-Tfh cells of tonsils (n = 4-7) as indicated in **(C)** are shown assessed by RT-qPCR analysis. GAPDH was used as a control. **(E)** Expression levels of granzyme A (GZMA) and granzyme B (GZMB)-positive cells in the DP-Tfh and SP-Tfh cells of tonsils (n = 4) as assessed by flow cytometry. **(F)** Expression level of CD70 in DP-Tfh and SP-Tfh cells of tonsils (n = 4) as examined by RT-qPCR analysis (left). FACS analysis of CD70 expression in these cells (middle, representative profiles, and right). **(G)** Expression level of CCL20 in DP-Tfh and SP-Tfh cells of tonsils as examined by RT-qPCR analysis (tonsil, n = 4; left). Expression of the CCL20 receptor, CCR6, on memory B cells assessed by flow cytometry (tonsil, n = 5; right). Data in **(A, D–G)** were analyzed by the Mann–Whitney U test.

To better understand the physiological characteristics of DP-Tfh cells, we conducted a comparative transcriptome analysis of tonsillar Tfh cell populations including DP-Tfh cells and single-positive (SP) GC-type Tfh cells (CD3^+^CD4^+^CD8^-^CXCR5^hi^PD-1^hi^, SP-Tfh cells) used as a control. DP-Tfh cells were detected in the tonsils of young (e.g., adenoid) and adult patients (**Figures 3A**, **5A**), whereas SP-Tfh cells were consistently found in the tonsils irrespective of age. Next, we investigated the transcriptomes of pairs of DP-Tfh and SP-Tfh cells derived from each individual tonsil (**Supplementary Table S6**). The results showed that DP-Tfh cells preferentially expressed transcripts related to CTLs, such as CD8 (CD8A and CD8B), Eomes, CRTAM, FAS ligand (CD95L), granzymes, and SLAMF7, indicating a possible cytotoxic attribute of DP-Tfh cells (**Figures 3B–E**). Th1 cell-related signature genes were also expressed in DP-Tfh cells, and cytokines, such as interferon (IFN)-γ and interleukin (IL)-10, were highly expressed in DP-Tfh cells rather than SP-Tfh cells. Of note, expression profiles of authentic GC-type Tfh (i.e., SP-Tfh)-related genes, such as IL-4, IL-21, Bcl6, and Pou2af1, appeared to be shared with DP-Tfh cells (**Figures 3C, D**). Among genes that regulated the interaction of T cells with B cells, DP-Tfh cells expressed the costimulatory molecule CD70 on their cell surface (**Figures 3C, F**). CD70 is a binding partner of CD27, which is highly expressed on class-switched B cells such as memory B cells, and the CD27/CD70 interaction usually facilitates memory B cells to differentiate into antibody-secreting cells (21, 22). CCL20 was highly expressed in DP-Tfh cells, but not in SP-Tfh cells, which is a ligand for the CCR6 presented on memory B cells (**Figure 3G**). These data suggest the possible engagement of DP-Tfh cells and memory B cells in lymphoid tissues (23).

### DP-Tfh cells exert a regulatory effect on memory B cells

**Figure 4:**
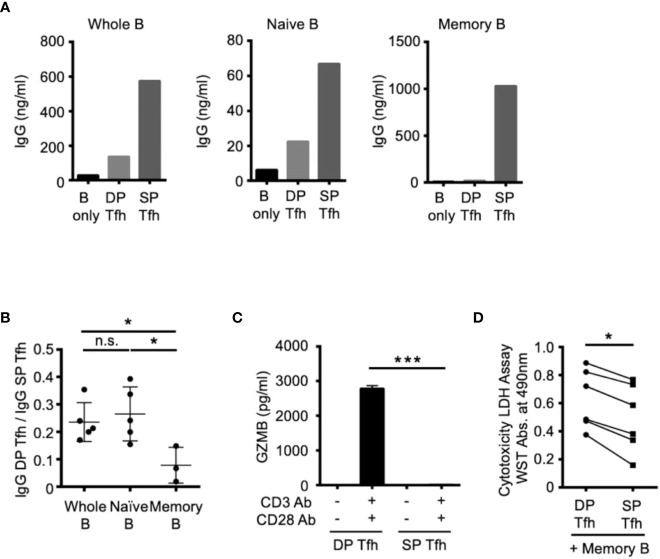
Functional effects of DP-Tfh cells on B-cell regulation. **(A)** Representative graphs of co-culture experiments to examine antibody production using autologous Tfh and B cells sorted from tonsil specimens (n = 3-5). DP-Tfh cells (CD3^+^CD4^+^CD8^+^CXCR5^hi^PD-1^hi^) or SP-Tfh cells CD3^+^CD4^+^CD8^-^CXCR5^hi^PD-1^hi^) were co-cultured with whole B cells (CD3^-^CD19^+^), naïve B cells (CD3^-^CD19^+^IgD^+^CD27^-^), or memory B cells (CD3^-^CD19^+^IgD^-^CD27^+^) under the stimulation of CD3, CD28, and CD40L. After incubating cells for 7 days, IgG levels in the supernatants were analyzed by ELISA. **(B)** Effects of DP-Tfh cells on B cells investigated by co-culture experiments as demonstrated in **(A)**. Data indicate ratios of IgG levels from whole B cells, naïve B cells, or memory B cells in the presence of DP-Tfh cells to those in the presence of SP-Tfh cells (IgG DP-Tfh/IgG SP-Tfh). Data were obtained from three to five independent experiments using autologous tonsillar lymphocytes. **(C)** Increased capacity of DP-Tfh cells to secrete granzyme B (GZMB) under CD3 and CD28 stimulation in comparison with SP-Tfh cells of tonsils. After incubating cells for 7 days, GZMB levels in culture supernatants were analyzed by ELISA. Data were obtained from four independent experiments using autologous tonsillar lymphocytes. **(D)** Cytotoxicity of DP-Tfh cells for memory B cells in comparison with SP-Tfh cells. Co-culture supernatants of memory B cells and DP-Tfh cells or SP-Tfh cells derived from autologous tonsillar lymphocytes were analyzed by a cytotoxicity LDH/WST assay. The absorbance values indicating the cytotoxic activities of DP-Tfh and SP-Tfh cells in each experiment (depicted as a closed circle and rectangle, respectively) are connected by a line to evaluate their differences. Data were obtained from six independent experiments. Statistical significance in **(B–D)** was determined by the Mann–Whitney U test.

We next investigated the specific cellular effects of DP-Tfh cells on B-cell subsets by co-culture experiments using autologous tonsillar lymphocytes. After T cell activation using anti-CD3 and anti-CD28 Abs, the SP-Tfh cells stimulated whole B cells and B-cell subsets, including naïve B cells and memory B cells, to produce antibodies (**Figure 4A**). In contrast to SP-Tfh cells, DP-Tfh cells induced a weaker antibody-producing effect in whole B cells and naïve B cells, but not memory B cells (**Figure 4A**). This was also indicated by the results from further experiments that analyzed the ratio of the IgG level from B cells induced by DP-Tfh cells to that of the IgG level from B cells induced by SP-Tfh cells (IgG DP-Tfh cells/IgG SP-Tfh cells, **Figure 4B**). The corresponding ratios for whole B cells and naïve B cells were comparable, whereas the ratio for memory B cells was markedly lower than that of whole and naïve B cells (**Figure 4B**). Together with evidence showing that DP-Tfh cells indeed secreted the cytotoxic molecule of granzyme B after CD3 and CD28 stimulation (**Figure 4C**), these data suggested that DP-Tfh cells induced memory B-cell death. After co-culture experiments of memory B cells and DP-Tfh cells or SP-Tfh cells as a control, a cytotoxic assay to measure lactate dehydrogenase (LDH) in the supernatants suggested that the regulatory activities of DP-Tfh cells strongly influenced memory B-cell fate (**Figure 4D**). Collectively, these results imply that DP-Tfh cells can act as an unidentified regulator of memory B-cell responses.

### Eomes^hi^CD70^hi^ DP-Tfh cells in lesions of IgG4-RD

**Figure 5:**
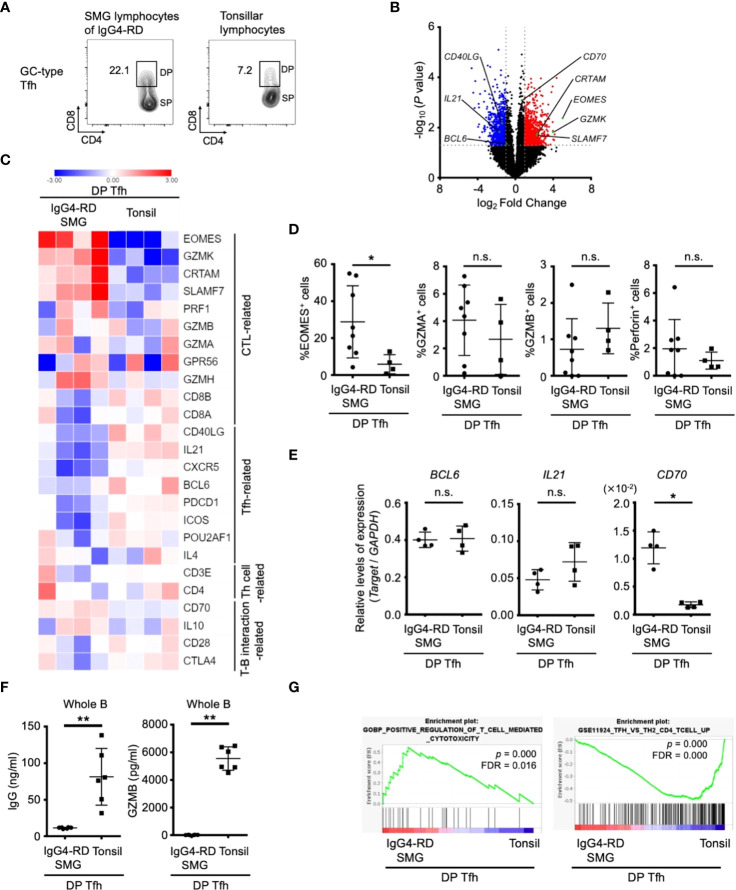
Features of DP-Tfh cells in inflammatory lesions of IgG4-RD. **(A)** Representative flow cytometry profiles of DP-Tfh cells and SP-Tfh cells in GC-type Tfh cells in the lymphocytes of tonsils and SMG lesions of IgG4-RD. **(B)** Volcano plot showing differentially expressed genes (*p* < 0.05) with more than two-fold expression in DP-Tfh cells localized in SMG lesions of IgG4-RD versus those in tonsils. Red and blue dots indicate the upregulated and downregulated genes, respectively, in the DP-Tfh cells of IgG4-RD. Data were obtained from microarray analysis of four specimens in each experimental group. **(C)** Heat map representing the relative abundances of transcripts identified in **(B)** for selected genes involved in the functioning and regulation of helper CD4^+^ T cells and CTLs. Relative values of gene expression are indicated by color. **(D)** Expression of CTL-related molecules in DP-Tfh cells of SMG lesions of IgG4-RD (n = 8) and tonsils (n = 4) as assessed by flow cytometry. **(E)** Relative levels of gene expression in DP-Tfh cells of SMG lesions of IgG4-RD (n = 4) and tonsils (n = 4) as indicated in **(B)** analyzed by RT-qPCR. GAPDH was used as a control. **(F)** Lower B-cell helper capacity of DP-Tfh cells in IgG4-RD lesions than in tonsils. The levels of IgG and granzyme B (GZMB) in supernatants from co-cultures of DP-Tfh cells with autologous whole B cells (CD3^-^CD19^+^) under stimulation of CD3 and CD28 were analyzed by ELISA on day 7 after initial incubation (IgG4-RD, n = 6; tonsil, n = 6). **(G)** Gene set enrichment analysis (GSEA) of genes identified in **(B)** showing transcriptomes of DP-Tfh cells. Results from gene sets associated with cytotoxic T cells or Tfh cells are shown on the left and right, respectively. Data in **(D–F)** were analyzed by the Mann–Whitney U test.

To further characterize the features of DP-Tfh cells in lymphoid tissues, we investigated the transcriptomes of DP-Tfh cells in SMG lesions of IgG4-RD in comparison with those of DP-Tfh cells in tonsils (**Figure 5A**). The results showed that DP-Tfh cells in IgG4-RD lesions expressed more CTL-related genes, such as Eomes and granzymes, than DP-Tfh cells in tonsils (**Figures 5B–D**, **Supplementary Table S7**). Notably, the CD70 level of DP-Tfh cells in IgG4-RD lesions was higher than that of tonsillar DP-Tfh cells (**Figure 5E**). Genes related to Tfh cell functions appeared to be expressed in DP-Tfh cells of tonsils rather than in DP-Tfh cells of IgG4-RD lesions (**Figure 5C**). DP-Tfh cells in tonsils potentially promoted IgG production from B cells, albeit with lesser ability than SP-Tfh cells, whereas DP-Tfh cells in IgG4-RD lesions did not (**Figures 4A–B**, **5F**). Considering the results of GSEA, DP-Tfh cells in IgG4-RD lesions may show greater cytotoxic capability than DP-Tfh cells in tonsils (**Figure 5G**). Taken together, these findings indicate that IgG4-RD lesions favorably contained DP-Tfh cells expressing Eomes and CD70 at high levels (Eomes^hi^CD70^hi^ DP-Tfh cells), with a high capacity to regulate memory B cells. Because Eomes and CD70 are usually upregulated in activated T cells in inflammatory tissues (24), they are also considered to be signature molecules of DP-Tfh cells induced during the persistent inflammation of IgG4-RD lesions. In our experiments, we could scarcely detect DP-Tfh cells in peripheral blood specimens of the participants (data not shown).

### SP-Tfh cells are the possible origin of DP-Tfh cells

**Figure 6:**
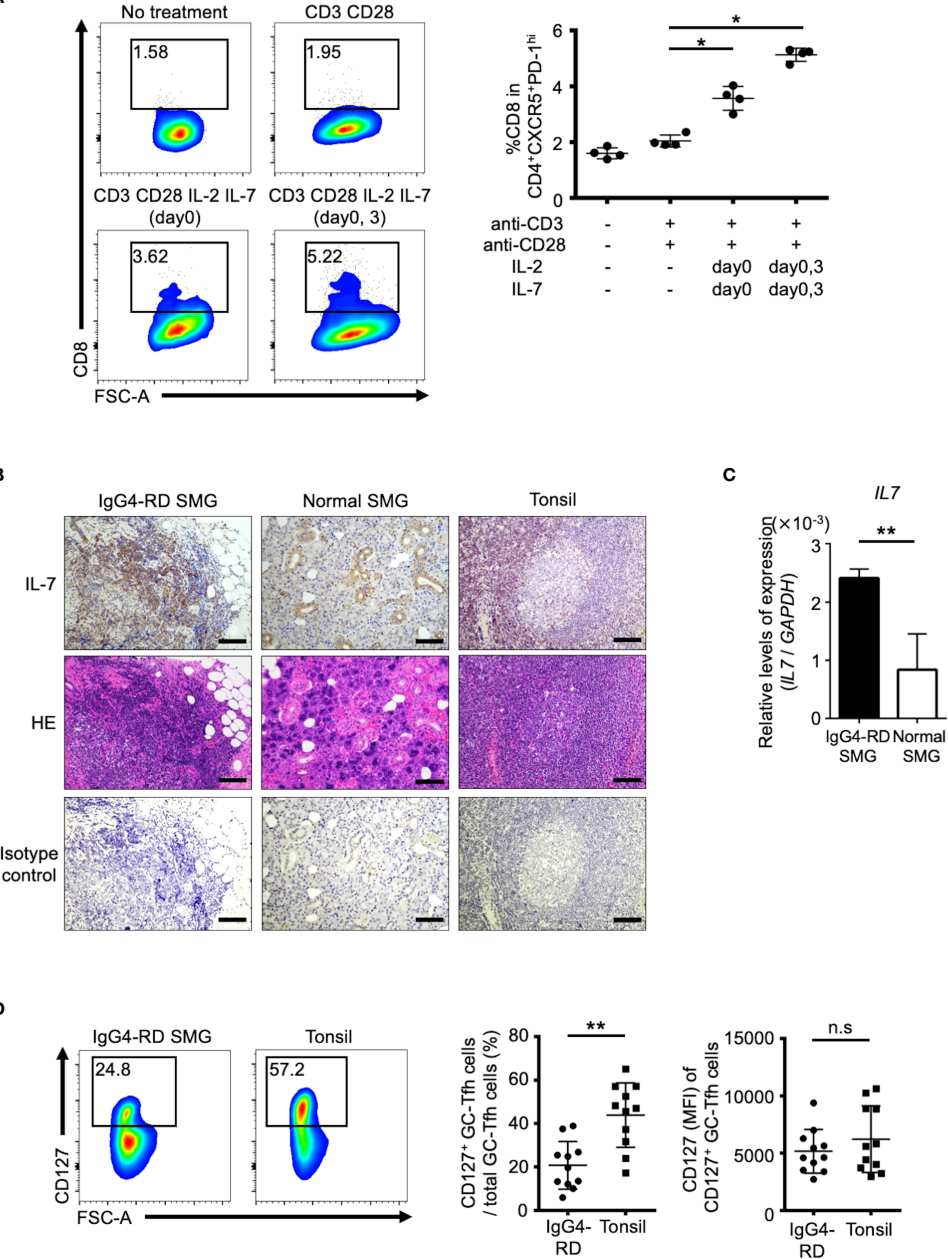
SP-Tfh cells are the possible origin of DP-Tfh cells. **(A)** Upregulation of CD8 on SP-Tfh cells (CD3^+^CD4^+^CD8^-^CXCR5^hi^PD-1^hi^) under the stimulation of IL-2 and IL-7. SP-Tfh cells (5×10^4^ cells/well) from tonsils (n = 4) were incubated in 200 μL of AIM-V medium with or without 2 μg/mL anti-CD3 and 2 μg/mL anti-CD28 mAbs. Under the stimulation of CD3 and CD28, 20 ng/mL IL-2 and 20 ng/mL IL-7 were added once on day 0 or twice on days 0 and 3. On day 7, cells were analyzed by flow cytometry. Representative flow cytometry profiles are shown on the left. Data obtained from four independent experiments are summarized in a graph on the right. **(B)** Histological examination of IL-7 expression. Immunohistochemistry of IL-7 in SMG lesions of IgG4-RD, normal SMG tissues, and tonsils are shown in the upper panels. The HE-stained images and isotype controls for immunohistochemistry in the corresponding tissue areas are shown in the middle and lower panels, respectively. Scale bar: 100 μm. **(C)** Relative expression levels of IL-7 in SMG lesions of IgG4-RD (n = 4) and normal SMG (n = 4) assessed by RT-qPCR analysis. GAPDH was used as a control. **(D)** Representative flow cytometry profiles showing the presence of CD127 on GC-type Tfh cells (CD3^+^CD4^+^CXCR5^hi^PD-1^hi^) in IgG4-RD lesions of SMGs and tonsils (left). The percentages of CD127^+^ GC-type Tfh cells among the total GC-type Tfh cells in IgG4-RD lesions and tonsils (right). Values of the mean fluorescence intensity (MFI) of CD127 on GC-type Tfh cells in the two groups are also shown (IgG4-RD, n = 11; tonsil, n = 11). Data in **(A, C, D)** were analyzed by the Mann–Whitney U test.

**Figure 7:**
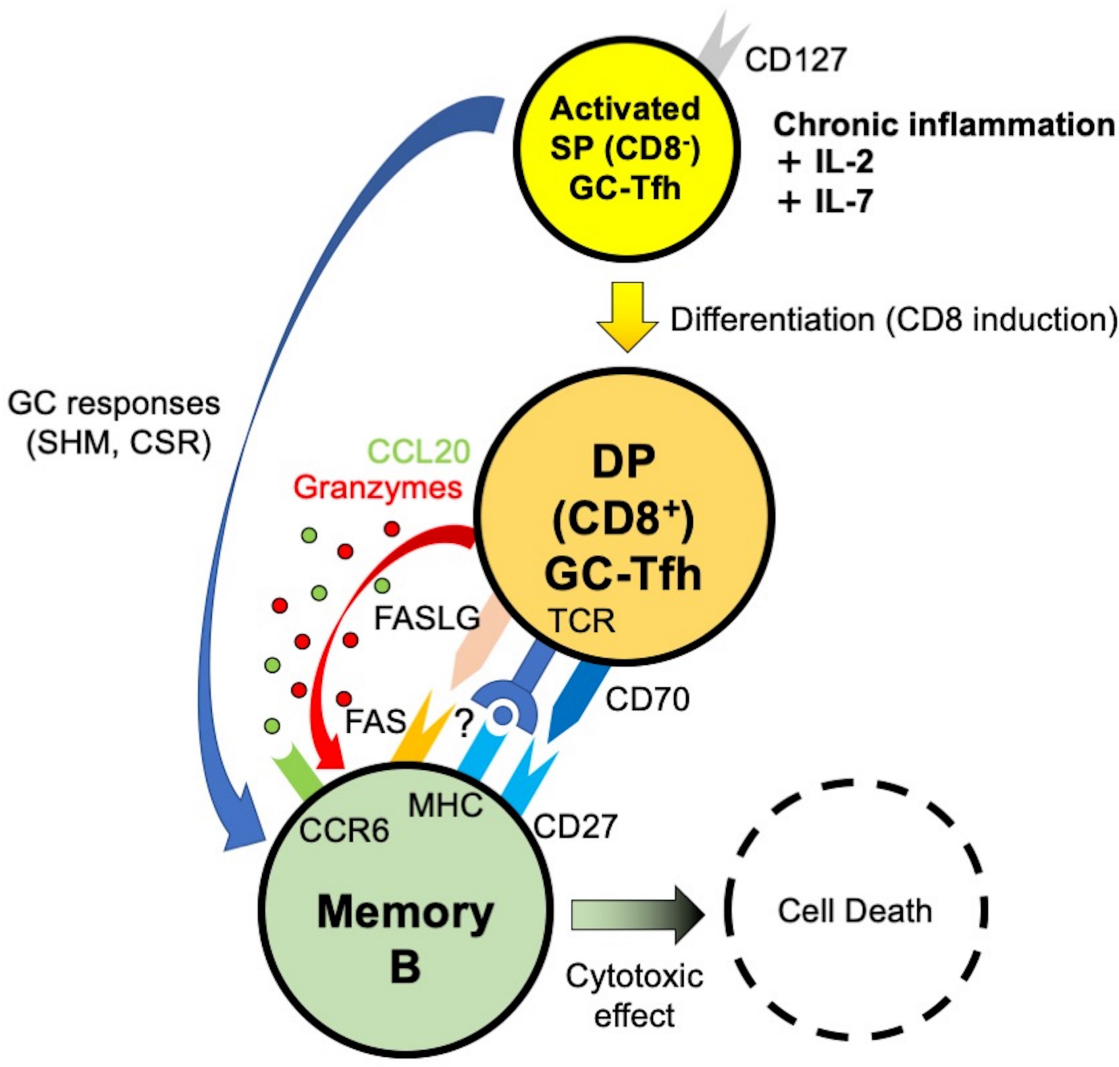
Model of DP-Tfh cell function in chronic inflammatory lesions of IgG4-RD. SHM, somatic hypermutation. CSR, class switch recombination.

Finally, we investigated the possible origin of tissue-resident DP-Tfh cells. In pathway analysis of upstream genes identified by transcriptome analyses of GC-type Tfh cells in IgG4-RD lesions and tonsils (**Figures 1B**, **3B**), a series of cytokines, including IL-2, IL-1β, CCL2, and IL-10, were postulated to be possible driver molecules related to the phenotype of lesional Tfh cells in IgG4-RD (**Supplementary Table S8A**). Further pathway analysis of transcriptomes in DP-Tfh cells and SP-Tfh cells of tonsils revealed that cytokines such as IL-2, IL-7, TNF, IL-6, IL-3, IL-1β, and IL-10 were involved in the maintenance of tonsillar DP-Tfh cells (**Supplementary Table S8B**). The gene expression profile of DP-Tfh cells was partly shared by authentic CD4^+^ Tfh cells (SP-Tfh cells) in tonsils; therefore, we performed *in vitro* analysis of tonsillar SP-Tfh cells under stimulation with different combinations of these cytokines. The results showed that consecutive stimulation by IL-2 and IL-7 efficiently induced CD8 expression in SP-Tfh cells (**Figure 6A**). IL-7 expression was highly detected in the inflammatory SMG lesions of IgG4-RD (**Figures 6B, C**). A receptor complex specifically bound to IL-7 (IL-7 receptor) is a heterodimer of the IL-7 receptor α chain (CD127) and common γ chain, which regulates T cell activation (25). We examined CD127 expression in DP-Tfh and SP-Tfh cells and found that the level of CD127^+^ Tfh cells in IgG4-RD lesions was relatively lower than that in tonsils (**Figure 6D**). However, we observed no significant differences in the mean fluorescent intensity (MFI) of CD127 (**Figure 6D**). Despite the primary structural differences in tonsils and inflamed SMGs of IgG4-RD lesions, which are secondary and tertiary lymphoid tissues, respectively, the lesions in IgG4-RD and tonsils seemed to contain CD127^+^ Tfh cells ready to receive IL-7. Therefore, SP-Tfh cells may be a potential origin of DP-Tfh cells in IgG4-RD lesions and tonsils. On the bases of the data obtained in this study, we proposed a model for DP-Tfh cells in the regulation of B cells in IgG4-RD lesions (**Figure 7**).

## Discussion

In this study, we describe CD4^+^CD8^+^ DP-Tfh cells that were defined as GC-type Tfh cells with possible cytotoxic activity to regulate the function of memory B cells in chronic inflammatory lesions. This follows the findings of a previous study that showed the abundance of tissue-resident Tfh cells in IgG4-RD lesions (13). Our findings are probably consistent with recent reports, which suggested the active involvement of cytotoxic CD4^+^ T cells in the pathogenesis of IgG4-RD (26, 27). Our study also suggests a potential transition of SP-Tfh cells to DP-Tfh cells under stimulation by the common γ-chain cytokines of IL-2 and IL-7. In line with this, GC-type Tfh cells, especially under persistent inflammation, coordinate humoral immunity by instructing a wider range of B cells than previously thought. IL-2 primes the activation of effector T cells and induces the expression of CD127 to enable T cells to bind to IL-7 for the formation of the memory T cell pool (25, 28). In addition to their general importance in the functional modulation of effector T cells, IL-2 and IL-7 have well-recognized roles in influencing the fate of Tfh cells (29–31). IL-2 controls the gene expression profile of Tfh cells during their initial development, whereas IL-7 signaling represses the functional expression of Bcl6 and induces cell surface expression of CD70 and PD-1 for efficient interactions between Tfh and B cells. In our findings, the ratio of DP-Tfh cells expressing CD127 in IgG4-RD lesions was relatively lower than that in tonsils. This is probably due to the difference in the primary structures of tonsils and IgG4-RD lesions, which constitute secondary and tertiary lymphoid tissues, respectively. Thus, it is reasonable to consider the transition of excess SP-Tfh cells to DP-Tfh cells in response to aberrant IL-7 and IL-2 concentrations in inflammatory lesions (32). According to our study, the inflammatory milieu in tertiary lymphoid tissues is suggested to allow the generation of DP-Tfh cells. Thus, in such immune settings, adaptive immunity depending on memory B cells may be regulated by DP-Tfh cells. Since DP-Tfh cells efficiently target IgG4^+^ memory B cells within the tertiary lymphoid lesions of IgG4-RD, these cells might have a unique capability to regulate the resolution of pathological immune responses. This regulatory mechanism is postulated to be a previously unidentified mechanism of immune tolerance mediated by Tfh cells. In instances where IgG4^+^ memory B cells reside in the lymph nodes, spleen and/or bone marrow of the patients with IgG4-RD, the regulatory function of DP-Tfh cells in memory B cells might be inhibited due to the lack of access of DP-Tfh cells to these tissues. This probably leads to high levels of serum IgG4 in patients with the IgG4-RD in comparison with healthy subjects.

Tfh cells expressing perforin and granzymes, which characterize a cytotoxic function (cytotoxic Tfh cells), are frequently detected in hospitalized patients with coronavirus disease 2019 (33, 34). Notably, cytotoxic Tfh cells are negatively correlated with the serum level of antibodies bound to the SARS-CoV-2 spike protein, implying the cardinal role of cytotoxic Tfh cells in the production of antigen-specific antibodies (33). Cytotoxic Tfh cells are further suggested to target GC B cells within tissues of chronic tonsillitis caused by periodic infection with group A Streptococcus (35). While the expression level of CD8 in such cytotoxic Tfh cells remains unclear, memory B cells may be regulated by cytotoxic Tfh cells, such as DP-Tfh cells, during viral and bacterial infections. In our study, Eomes^hi^CD70^hi^ DP-Tfh cells in IgG4-RD lesions potentially showed a robust cytotoxic function in comparison with tonsillar Eomes^+^ DP-Tfh cells. Thus, the cell lytic activities of DP-Tfh cells appear to be associated with their expression level of Eomes, which is a homologous T-box transcription factor T-bet and establishes a cytotoxic effector profile of natural killer cells and CD8^+^ CTLs (16, 36). A recent study focusing on Eomes^+^CD4^+^ CTLs has expanded and documented their roles in anti-tumor immunity and pathological responses in inflamed conditions such as rheumatoid arthritis and multiple sclerosis (24, 37). Investigations focusing on the mechanisms regulating the expression of Eomes in DP-Tfh cells may improve our understanding of the functional significance of DP-Tfh cells in various immune settings. DP-Tfh cells of mice immunized with ovalbumin did not seem to be associated with the MHC Class I tetramer specific to ovalbumin, which could certainly bind to CD8 cytotoxic T cells (unpublished observation), suggesting that the involvement of MHC Class I molecules in DP-Tfh cell function may be minimal.

Polarized Tfh-cell subsets are identified among blood lymphocytes probably affected by the surrounding cytokine milieu, which influences the differentiation of the helper CD4^+^ T cells to distinct subsets (38). Conversely, the DP-Tfh cells are detected in tissues, but not in blood specimens, implying that their primary function may be limited to the local lymphoid tissues where they are produced. In our study of tonsillar lymphocytes, the percentages of cells expressing cytotoxic molecules like granzymes and perforin in non-Tfh DP cells were significantly lower than those of DP-Tfh cells (unpublished observation), further implying the cytotoxic capability of DP-Tfh cells in tissues. Peripheral extrathymic CD4^+^CD8^+^ T cells have been well studied across species (39). Human CD4^+^CD8^+^ T cells have been suggested to show an anti-tumor capacity that is hampered by the major histocompatibility complex molecules in tumor tissues (40). CD4^+^CD8^+^ T cells are known to include a subpopulation of cells sensitive to IL-2 and IL-7 with cell lytic enzymes, as seen in healthy subjects, thereby supporting the critical role of these cytokines in the regulation of DP-Tfh cells (41). CD8^+^ follicular T cells (CXCR5^+^PD-1^+^) are a subset of CD8^+^ T cells in mice and regulate GC B cells (42). A similar phenotype of CD8^+^ follicular T cells has also been reported in human blood and suggested to be involved in the pathogenesis of viral hepatitis and Sjögren’s syndrome (43, 44). Currently, the relationship between DP-Tfh and CD8^+^ follicular T cells remains elusive. Nonetheless, studies focusing on the diversity and plasticity of Tfh cells are warranted to illustrate the functional properties of Tfh cells in secondary and tertiary lymphoid tissues as well as in peripheral blood and thereby to improve our understanding of the pathogenesis of immune-related disorders.

In summary, we report DP-Tfh cells as a heterogeneous subpopulation of GC-type Tfh cells that are enriched in IgG4-RD lesions. DP-Tfh cells may regulate IgG4 production of memory B cells in IgG4-RD, and DP-Tfh cells are a potential target to improve the pathological immune settings of IgG4-RD. As an active interaction of GC B cells and SP-Tfh cells (CD4^+^ GC-Tfh cells) proceeds, further interaction of memory B cells and DP-Tfh cells may rationally lead to efficient regulation of humoral responses, especially in inflammatory conditions. This hypothesis may be further supported by evidence suggesting a similar expression level of CXCR4 orchestrating GC reactions in SP-Tfh and DP-Tfh cells of tonsils (unpublished observation). Considering the importance of Tfh cells in protective immune responses, future research based on the present study could also provide an efficient modality to induce vaccine-specific antibodies for preventing infections caused by harmful pathogens.

## Data availability statement

Microarray data in this manuscript are available from Gene Expression Omnibus repository hosted by the National Center for Biotechnology Information (accession numbers GSE202615, GSE202616, and GSE202617 for the data presented in **Figures 1B**, **3B**, **5B**, respectively). The original contributions presented in the study are included in the Supplementary Material. Further inquiries can be directed to the corresponding authors.

## Ethics statement

Experiments using clinical materials were approved by the Institutional Review Board of Sapporo Medical University Hospital (IRB#25-39, IRB#292-83). All participants provided written informed consent in accordance with the Declaration of Helsinki.

## Author contributions

KM, II, RK, and HS performed experiments and analyzed data. M. Yanagi, SK, and TS assisted with the experiments. AS, KS, M. Yamamoto, HT, and KT discussed data. SI designed the experiments and prepared the manuscript. All authors approved the final version of the submitted manuscript.

## Funding

This study was funded by Grants-in-Aid from the Japan Society for Promotion of Science (JSPS) to KM (#19K18776), II (#19K16614), RK (#21K09658), and SI (#18H02632).

## Acknowledgments

We thank all the participants for their support and advice.

## Conflict of interest

The authors declare that the research was conducted in the absence of any commercial or financial relationships that could be construed as a potential conflict of interest.

## Publisher’s note

All claims expressed in this article are solely those of the authors and do not necessarily represent those of their affiliated organizations, or those of the publisher, the editors and the reviewers. Any product that may be evaluated in this article, or claim that may be made by its manufacturer, is not guaranteed or endorsed by the publisher.

## Supplementary material

Supplementary Table 1 Study participant characteristics.

Supplementary Table 2 Types of organ involvement in patients with IgG4-RD.

Supplementary Table 3 List of antibodies for flow cytometry and immunohistochemistry, and reagents used for *in vitro* experiments.

Supplementary Table 4 PCR primers used in this study.

Supplementary Table 5 Differentially expressed genes between Tfh cells in IgG4-RD lesions and Tfh cells in tonsils assessed by microarray analysis.

Supplementary Table 6 Differentially expressed genes between DP-Tfh and SP-Tfh cells in tonsils assessed by microarray analysis.

Supplementary Table 7 Differentially expressed genes between DP-Tfh cells in IgG4-RD lesions and DP-Tfh cells in tonsils assessed by microarray analysis.

Supplementary Table 8 Genes selected by iPathwayGuide analysis to predict regulators of DP-Tfh cells based on the transcriptomes of GC-Tfh cells, including DP-Tfh cells and SP-Tfh cells. **(A)** IgG4-RD Tfh cells vs. tonsillar Tfh cells. **(B)** Tonsillar DP-Tfh cells vs. tonsillar SP-Tfh cells
